# Generalization versus Specialization in Pollination Systems: Visitors, Thieves, and Pollinators of *Hypoestes aristata* (Acanthaceae)

**DOI:** 10.1371/journal.pone.0059299

**Published:** 2013-04-10

**Authors:** Eliška Padyšáková, Michael Bartoš, Robert Tropek, Štěpán Janeček

**Affiliations:** 1 Faculty of Science, University of South Bohemia, České Budějovice, Czech Republic; 2 Institute of Botany, Academy of Sciences of the Czech Republic, Třeboň, Czech Republic; 3 Institute of Entomology, Biology Centre, Academy of Sciences of the Czech Republic, České Budějovice, Czech Republic; Universidad Nacional Autonoma de Mexico, Mexico

## Abstract

Many recent studies have suggested that the majority of animal-pollinated plants have a higher diversity of pollinators than that expected according to their pollination syndrome. This broad generalization, often based on pollination web data, has been challenged by the fact that some floral visitors recorded in pollination webs are ineffective pollinators. To contribute to this debate, and to obtain a contrast between visitors and pollinators, we studied insect and bird visitors to virgin flowers of *Hypoestes aristata* in the Bamenda Highlands, Cameroon. We observed the flowers and their visitors for 2-h periods and measured the seed production as a metric of reproductive success. We determined the effects of individual visitors using 2 statistical models, single-visit data that were gathered for more frequent visitor species, and frequency data. This approach enabled us to determine the positive as well as neutral or negative impact of visitors on *H. aristata’*s reproductive success. We found that (i) this plant is not generalized but rather specialized; although we recorded 15 morphotaxa of visitors, only 3 large bee species seemed to be important pollinators; (ii) the carpenter bee *Xylocopa* cf. *inconstans* was both the most frequent and the most effective pollinator; (iii) the honey bee *Apis mellifera* acted as a nectar thief with apparent negative effects on the plant reproduction; and (iv) the close relationship between *H. aristata* and carpenter bees was in agreement with the large-bee pollination syndrome of this plant. Our results highlight the need for studies detecting the roles of individual visitors. We showed that such an approach is necessary to evaluate the pollination syndrome hypothesis and create relevant evolutionary and ecological hypotheses.

## Introduction

Debates about the generalization or specialization of pollination systems have been a prevailing theme in pollination ecology for many years. During that time, the view has been that pollination systems permanently balanced on the specialization–generalization continuum [1]. The original idea that co-evolution often resulted in the specialization of plants and their pollinators came firstly up with Darwin’s evolutionary theory [2] and then was extended in later works [3]. The specialization has been discussed over a long period and is closely related to the concept of pollination syndromes [4]–[7], which are defined as a set of traits that convergently evolved as adaptations to similar pollinators. Simultaneously, the pollination syndrome concept has been opposed by some pollination biologists who noted that the links between floral traits and observed visitors are much weaker than predicted [8], [9] and that co-evolution is often diffuse [10]. Whereas the existence of generalized pollination systems was firstly manifested only for some plant species [11]–[13], the more recent community-wide studies have shown that flowers of most plants are visited by a relatively high diversity of visitors and that generalization is much more common than was previously expected [14]–[18].

Nevertheless, this broad generalization hypothesis has been criticized by other researchers [1], [19], [20] who argue that some floral visitors that are usually considered in pollination webs are actually ineffective pollinators. In fact, a broad spectrum of diverse floral visitors with positive, neutral, and even negative effects on plant reproductive success can be found [21]–[24]. Several different techniques can be used to test the effects of particular pollinators. Indirect techniques, such as estimating visitor frequency rates [25]–[27] or direct measuring the total amount of pollen grains brought onto the stigma during a single visit of a particular visitor [25], [26], [28]–[31], may not sufficiently consider the real contribution of particular visitors to the plant’s reproduction [32]. One possible way to detect the visitor’s actual contribution directly is by using estimates from single visits to virgin flowers [33]–[35]. However, the single-visit approach has several weaknesses. Although it allows positive contributions to plant reproduction (i.e. the contribution of effective pollinators) to be quantified, it is not possible to reveal any negative effects of other visitors, so those visitors are simply classified as ineffective pollinators. Since many studies have shown negative effects of floral visitors [36]–[38], these should be considered whenever hypotheses on floral evolution are developed [36].

Here, we focus on the pollination system of a broadly distributed Afrotropical plant species, *Hypoestes aristata*. This species shows the pollination syndrome [4], [39] associated with bee pollination. Typically, its flowers have nectar-guide markings and produce a small amount of highly concentrated nectar. However, according to previous studies it is visited by a much broader spectrum of potential pollinators, including long-proboscid flies in South Africa [40], [41] and various sunbirds, bees, flies, butterflies, and moths in our study area in the Bamenda Highlands, Cameroon [42], [43]. In this area, *H. aristata* is the most favoured food plant of the sunbird *Cinnyris reichenowi* [44]. Although the *H. aristata* morphology suggests pollinator specialization, it is apparently visited by a variety of birds and insects. Thus, *H. aristata* is an ideal model plant species for testing the validity of the concept of pollination syndromes. Simultaneously, examining its pollination system can contribute to the current debate about the proportion of generalization and specialization in pollination biology. The aim of our study was to answer the following main questions: (1) What is the spectrum of floral visitors of *H. aristata*? (2) Which visitors are effective pollinators? (3) Which visitors have neutral or negative effects on the reproduction of *H. aristata*? (4) Is the pollination system of *H. aristata* rather generalized, as suggested by previous studies on its floral visitors, or more specialized, as predicted by its floral traits? and (5) Is the bee pollination syndrome a good predictor of effective pollinators?

## Methods

### Study Site

The study site was situated in the Mendong Buo area (6°5′26′′N 10°18′9′′E; 2100–2200 m a.s.l.), ca. 5 km southeast from Big Babanki (Kedjom-Keku community), in the Bamenda Highlands, North-West Province, Cameroon. This area is a mosaic of extensive pastures, frequently burned forest clearings dominated by *Pteridium aquilinum*, shrubby vegetation along streams, and remnants of species-rich tropical montane forests with a frequent occurrence of *Schefflera abyssinica*, *Schefflera manii*, *Bersama abyssinica*, *Syzygium staudtii*, *Carapa procera*, and *Ixora foliosa*. There is a single wet season from March to November, with annual precipitation ranging from 1780 to 2290 mm/year (For more details see: Cheek et al., Reif et al. & Tropek et al. [45]–[47]).

Our research was permitted by the Ministry of Scientific Research and Innovations of the Republic of Cameroon (permit no. 93/MINRESI/B00/C00/C10/C12) and the Ministry of Forestry and Wildlife of the Republic of Cameroon (permit no. 2306/PRBS/MINFOF/SG/DFAP/SDVEF/SC). Voucher insects were exported with the permission of the Ministry of Agriculture and Rural Development of the Republic of Cameroon (permit no. 15347/A/PPP/LBE). Our research was also permitted by Benjamin Vubangsi, the local chief of the Kedjom-Keku community, which owns the study area. The study was not conducted in any of the protected areas or on any protected species.

### Plant Species

**Figure 1:**
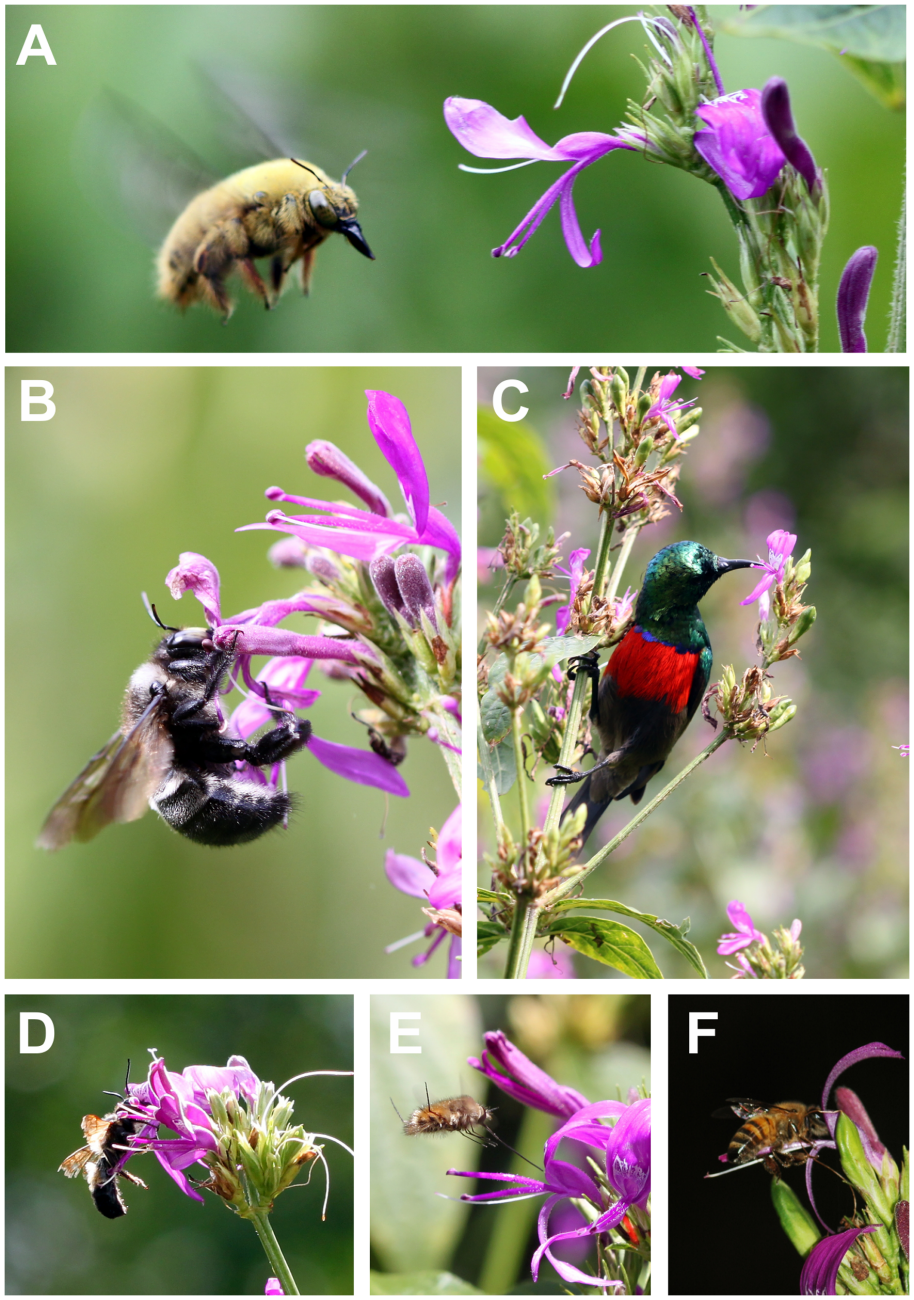
The visitors of *Hypoestes aristata*: (A) *Xylocopa* cf. *inconstans*; (B) *Xylocopa lugubris*; (C) *Cinnyris reichenowi*; (D) *Megachile* sp.; (E) *Bombyliidae*; (F) *Apis mellifera*. Photos (A)–(E) by R. Tropek, (F) by Š. Janeček.

Our target plant species, *Hypoestes aristata* (Vahl) Sol. ex Roem. & Schult var. *aristata* (family Acanthaceae), is a clonal herb that grows up to 1.5 m high and is native to tropical sub-Saharan Africa [48], [49]. The plant has hermaphroditic, zygomorphic flowers that are crowded into verticillate inflorescences. Dark purple blossoms with white nectar-guide markings on the upper lip have a pistil and 2 stamens long exerted from the corolla (Fig. 1). *H. aristata* produces a low volume (1.27 µl per flower) of hexose-dominant nectar of highly variable concentration (62.21% ±24.13; mean concentration ± [SD] w/w; i.e. sucrose equivalent mass/total mass; [43]). Nectar is accumulated in its 1-cm-long, narrow corolla-tube. Individual flowers last for about 5 days and can be found throughout the dry season. After pollination, a flower turns into a dehiscent capsule with up to 4 seeds (pers. obs.). *H. aristata* forms dense clumps, with several shoots flowering more or less simultaneously, which increases its local attractiveness for visitors. Usually, the plant dominates locally in disturbed montane forests, at their edges, in shrubby vegetation along streams, and in successionally older clearings. Experimental hand-pollinations during a preliminary study showed that *H. aristata* cannot effectively reproduce via autonomous selfing or parthenogenesis, and thus, is fully dependent on its pollinators (File S1; Fig. A in File S1; Table A in File S1).

### Flower Visitors and Pollination Effectiveness

The flower visitors were studied from November to December 2010, when the plants of *H. aristata* are in full bloom. Ten shoots in 10 patches of flowering *H. aristata* were chosen within the whole study area. Shoots with several target flower buds were bagged individually with a fine mesh and the buds were marked. The bags were large enough to allow the flowers to completely open inside the netting. The following day, all open marked flowers on a shoot (5.3±1.29 per shoot; mean ± standard deviation [SD]) were observed simultaneously for a 2-hour session (i.e. one shoot with several open flowers was observed in one session) and all flowers were bagged again immediately after the observation. During each observation session, all animals that visited the marked flowers were recorded and identified to morphotaxa (Table 1, Movie S1). Observations of individual shoots were equally distributed throughout the day (between 0700 and 1800) to include all possible diurnal visitors and were limited to suitable weather conditions (sunny or partly cloudy). Fruits were harvested after maturation and their seeds were counted and weighed.

**Table 1:** The effect of individual flower visitors on seed production in *H. aristata*.

			MODEL 1				MODEL 2				
			Marginal tests		Whole model		Marginal tests			Whole model	
Order	Family	Species	r	F	Es.	F	Pr.	Ab.	F	Es.	F
Passeriformes	Nectariniidae	*Cinnyris bouvieri*	0.030	0.49	–	–	0.75	0.36	1.24	0.003	1.78
		*Cyanomitra oritis*	0.044	1.06	0.011	1.91	0.80	0.36	3.03	–	–
		*Cinnyris reichenowi*	−0.093	4.73*	–	–	0.19	0.41	3.91	–	–
Diptera	Bombyliidae		−0.021	0.23	–	–	0.26	0.37	0.23	–	–
	Syrphidae		0.045	1.07	–	–	0.58	0.35	1.22	–	–
	Other dipterans		0.091	4.51*	0.002	1.82	0.36	1.14	4.51*	0.001	1.71
Lepidoptera			−0.033	0.58	–	–	0.00	0.37	0.58	–	–
Hymenoptera	Apidae	*Apis mellifera*	−0.084	3.77	0.010	3.22	0.21	0.42	4.73*	0.035	8.58**
		*Anthophora* sp.	0.036	0.70	–	–	0.52	0.36	0.79	–	–
	Megachilidae	*Megachile* sp.	0.089	4.71*	0.011	6.41*	1.00	0.36	4.31*	0.010	6.01*
	Other wild bees		−0.027	0.40	–	–	0.21	0.37	0.36	–	–
	Apidae	*Xylocopa* cf. *inconstans*	0.176	17.2**	0.040	10.64**	0.50	0.22	10.78**	0.010	4.00*
		*Xylocopa nigrita*	−0.028	0.41	–	–	0.00	0.37	0.43	–	–
		*Xylocopa erythrina*	−0.028	0.41	0.000	1.116	0.00	0.37	0.43	0.002	1.60
		*Xylocopa lugubris*	0.025	0.33	0.006	2.2228	0.39	0.35	0.21	–	–

Permutation models: ***Model 1*** assumed that visitors continuously saturate stigmas with pollen grains, i.e. the number of visits by individual visitors represented the explanatory variables. Marginal tests for this model represent individual regressions. ***Model 2*** is based on the idea that the flower received sufficient pollen to produce the maximum of seeds after one visit from a pollinator (i.e. visitor presence/absence data were used). Marginal tests represent the individual permutation ANOVAs. Abbreviations: **r,** Pearson correlation coefficient; **F,** F ratio; **Es.,** unbiased estimate of the components of variation, which shows the relative importance of individual terms in the model in relation to overall variation; **Pr.,** mean number of seeds which developed from flowers visited at least once by the visitor; and **Ab.,** mean number of seeds which developed from flowers not visited by the visitor. Significant differences (*0.01<p<0.05; **p<0.01) are in bold. The results for the random term ‘shoot’, which were always significant, were included in the whole models but are not presented. For more details, see Methods.

### Statistical Analyses

Due to many zero values, the data on seed production were not normally distributed. We thus analysed the effects of particular flower visitors on seed production using non-parametric permutation models. Seed numbers produced by individual flowers served as a dependent variable and visits of individual visitors as explanatory variables (i.e. each visitor represents one explanatory variable in each analysis). These explanatory variables contained either abundance data (i.e. numbers of visits to individual flowers during 2-hour observations – see Model 1 below) or presence-absence data (i.e. the information if the visitor at least once visited or did not visit the flower – see Model 2 below). Note that we also considered the value of zero at the unvisited flowers for abundance data in Model 1. To avoid the variability in seed production that can be explained by having more than one visitor to a flower during the 2-hour session we used the Type II sums of squares approach for a given explanatory variable [50]–[52]. In this way, the sum of squares for each visitor (explanatory variable) was calculated as the increase of the model sum of squares (and equivalently the decrease in the error sum of squares) due to adding this visitor into a model that already contained all of the other visitors [50]. Thus, only the variability that could not be explained by other than just the tested visitor was considered. Two models with different biological predictions were established. Model 1 assumed that the number of developed seeds increases or decreases with visitation frequency (e.g. visitors continuously saturate the stigma with pollen grains or continuously consume nectar from the flower and decrease the attraction of the flower by this way). Model 2 assumed that the most important is whether the visitor visit the flower or not (e.g. flower receives enough pollen to produce the maximum number of seeds after a single visit from each pollinator or the nectar is completely depleted during the single visit). Following these approaches, the log (x+1) transformed numbers of visits by individual visitors to each flower were used in the first model as an explanatory variable, whereas binary coded visits (i.e. at least one visit = 1, no visit = 0) to each flower were analysed in the second model. In both the models, the factors (visitors) with high p-values and negligible contribution to total variation in seed set among flowers indicated by negative estimates of the component of variation were stepwise excluded from the model [51], [53], [54]. After exclusion of the term with the lowest negative value of the component of variation, the models were recalculated. Consequently, only visitors with positive values of components of variance remained in the models [53]. The spatial autocorrelation effect (i.e. the term ‘shoot’) was considered in the models as a random variable. This term was always significant (i.e. individual shoots differed), and we have not shown the results for this term in Table 1. Except for the above described whole models, where all visitors were considered, we calculated marginal tests for each of the visitors. These tests demonstrate how visits of each visitor are related to seed production when each visitor is taken alone, ignoring others. Permutation tests were run with PERMANOVA+ for PRIMER [53].

## Results

During the observations of 539 flowers, 1979 flower visits, involving fifteen visitor morphotaxa, were recorded (Table 1). On average, 198 (±68.52) visits per patch and 3.67 (±2.61) visits per flower were detected. Although more than 95% of the flowers were visited at least once, less than 15% of the visited flowers produced fruit with viable seeds.

**Figure 2:**
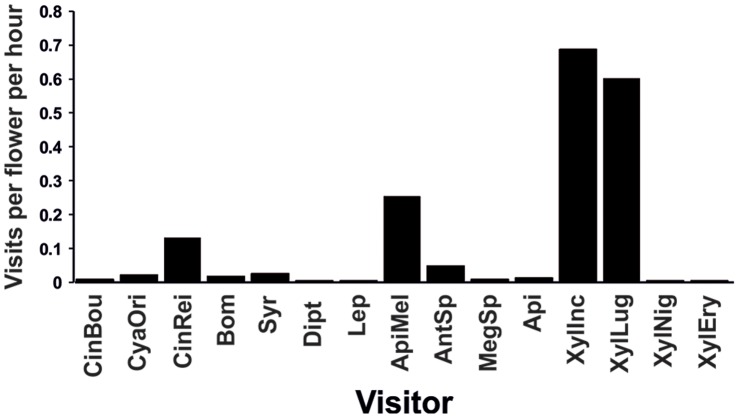
Total visitation frequencies. Abbreviations: **CinBou** = *Cinnyris bouvieri*, **CyaOri** = *Cyanomitra oritis*, **CynRei** = *Cinnyris reichenowi*, **Bom** = Bombyliidae, **Syr** = Syrphidae, **Dipt** = other dipterans, **Lep** = Lepidoptera, **ApiMel** = *Apis mellifera*, **AntSp** = *Anthophora* sp., **MegSp** = *Megachile* sp., **Api** = other bees, **XylInc** = *Xylocopa* cf. *inconstans*, **XylLug** = *Xylocopa lugubris*, **XylNig** = *Xylocopa nigrita*, **XylEry** = *Xylocopa erythrina*.

The total visitor community was highly dominated by two carpenter bees: *Xylocopa* cf. *inconstans* (Fig. 1A; including *X. inconstans* and *X. caffra*, which are hardly recognisable from each other in the field) and *Xylocopa lugubris* (Fig. 1B); followed by the honeybee *Apis mellifera* (Fig. 1F) and the northern double-collared sunbird *Cinnyris reichenowi* (Fig. 1C; Fig. 2). Nevertheless, the visitors’ abundances and community composition differed considerably among patches (Figure S1). All the studied patches had a similar pattern of visitor distribution, with one or a few highly abundant taxa, while most other visitors were rarely observed. *X. lugubris* was the only visitor taxon observed at all studied patches.

Although 5 visitor taxa significantly affected seed production, if the other visitors were not considered (marginal tests for models 1 and 2, Table 1), only three visitor taxa were able to explain the variability in the reproductive success of *H. aristata* when the variability which could be explained by more visitors was eliminated (whole models 1 and 2, Table 1). Both the whole models indicated that the carpenter bee *X.* cf. *inconstans* and the leafcutter bee *Megachile* sp. (Fig. 1D) increased plant reproductive success, whereas the honeybee *A. mellifera* was related to fruit abortion (Table 1). According to the estimated values in the first model, *X.* cf. *inconstans* is three times more important pollinator than *Megachile* sp. Most of the variability in the second model was explained by the visits of *A. mellifera*.

The majority of the flowers were visited repeatedly during our observations, usually by more than one visitor taxon, but 79 observed flowers were visited just once. These single visits were made by the four most frequent visitors, but flowers produced seeds only after a single visit of either *X.* cf. *inconstans* or *X. lugubris*, not of *A. mellifera* or *C. reichenowi* (Table 2). Although the flowers visited once by these four visitors did not significantly differ in seed production (permutation ANOVA; d.f. = 3; F = 1.98; p = 0.114), *Xylocopa* spp. differed from *A. mellifera* and *C. reichenowi* which were indicated by the models (Table 1) as visitors with rather negative influence on the seed production (permutation ANOVA; d.f. = 2; F = 5.07; p = 0.039). Although a honeybee might receive a pollen load from the anthers, it rarely deposits the pollen because it is too small to touch the stigma when inserting its head into the flower to forage on nectar (see Fig. 1F). Similarly, sunbirds, while visiting, introduced their bills partially or totally into the floral tube in a space between the upper lip and both sexual organs.

**Table 2:** List of visitors with more than 5 single-visits, and the mean number of seeds ± standard deviation (SD) for each flower.

Visitor	Number of single-visits	Seeds/visit
***Xylocopa*** ** cf. ** ***inconstans***	22	0.455±1.06
***Xylocopa lugubris***	21	0.238±0.70
***Apis mellifera***	14	0±0
***Cinnyris reichenowi***	13	0±0

Summarizing all the analyses performed, the carpenter bee *X.* cf. *inconstans* seemed to be the main pollinator of the plant in the study area. The importance of the other carpenter bee, *X. lugubris,* followed from its total high frequency of visits. *X. lugubris* equally visited the successfully and unsuccessfully pollinated flowers, which means that, in total, it contributed to pollination of the flowers only occasionally. Its high frequentness, however, guarantees a relatively bigger contribution to seed production than the less frequent visitors. The leafcutter bee *Megachile* sp. positively affected seed production of *H. aristata* (Models I and II in Table 1), nevertheless its visitation rate was too low (Fig. 2) to be crucial to *H. aristata*’s reproduction in the study area.

## Discussion

We have described the reproductive and pollination system of *H. aristata,* and have shown that the apparently generalized pollination system is actually highly specialized in the study area and that the effective pollinators are in agreement with the pollination syndrome of this plant.

Due to our experimental approach, we were able to determine not only the pollinator effectiveness but also the negative impact of visitors on the studied plant’s reproduction. Interestingly, single visits from 2 frequent visitors, the honeybee *A. mellifera* and the sunbird *C. reichenowi*, did not result in any seed production, and visits of *A. mellifera* even decreased the reproduction success of *H. aristata*.

The effectiveness of both the above mentioned carpenter bees in the *H. aristata* pollination system is in accordance with statements of other researchers, showing the *Xylocopa* species as extremely important pollinators in various tropical systems [55]–[57]. The honey bee *A. mellifera* is commonly considered to be a generalist forager, visiting many plant species [58]. Although it usually visits flowers more frequently than other flower visitors [59]–[61], its effectiveness as a pollinator is likely to differ, depending upon its foraging behaviour [59], [62] and the morphology of the flowers [60]. Our finding that *A. mellifera* had a negative impact on *H. aristata* seed production might be because of a combination of both of the above-mentioned factors. We assume that, as has been shown by other studies [62], [63], *A. mellifera* acted as a floral thief, removing a substantial part of the available nectar or pollen and thus making the flower unattractive for other visitors.

Among the three sunbird species visiting *H. aristata*, *C. reichenowi* was the most frequent visitor [42], [44], but it did not effectively pollinate the flowers. Its ineffectiveness could be related to the relatively small and specialized flowers of *H. aristata* that do not fit the birds’ heads (Fig. 1C). Thus, the anthers and stigma contacted the lower part of the bird’s bill, which seems to be inappropriate for pollen transfer. In bird-pollinated flowers, pollen grains typically attach firmly to a bird’s crown when the bird inserts its bill into the perianth to extract nectar [64], [65]. On the basis of our results, we consider *C. reichenowi* to be a nectar thief, although there was no obvious negative effect on *H. aristata* reproduction, in contrast to that by *A. mellifera*. In accordance with our observations (Fig. 1), we agree that ‘trait-matching’ between flowers and their visitors plays an important role in pollination interactions [24], [44], [66]–[68].

A limitation of our study is the relatively small study area size and short time in which the study was performed. It has been shown that diversity, abundance, and the importance of individual visitors may differ depending on the time and place [69]–[72]. Conversely, *H. aristata* in South Africa is also visited by carpenter bees [40]; thus, there is a high possibility that they are the main pollinators in that region. Moreover, our findings are in accordance with the expectations from ‘trait-matching’; i.e. the honeybee *A. mellifera* rarely reaches the stigma to deposit pollen and the sunbird carries pollen on its lower bill. Therefore, neither of these species should be an effective pollinator. Nevertheless, similar studies conducted in different African regions would substantially contribute to this debate.

Choosing the right field technique for measuring the pollination or plant reproductive success is important since there are several possible methods with various weaknesses and benefits [32]. Because of the shortcomings of using the single-visit method to estimate pollination effectiveness [33], [35], we chose the approach based on 2-hour observation periods. Basing observations on time-defined periods is more suitable to detect the potential effects of the whole spectrum of floral visitors, including occasional visitors; and to reveal both positive and negative effects of individual visitors. This method is, moreover, less laborious than bagging flowers after each single visit. If the length of the observation period is well chosen the dataset can also include single-visit data, at least for the more frequent pollinators. A drawback of this method follows the fact that the seed set is usually formed after multiple visits from the same or different visitors.

**Figure 3:**
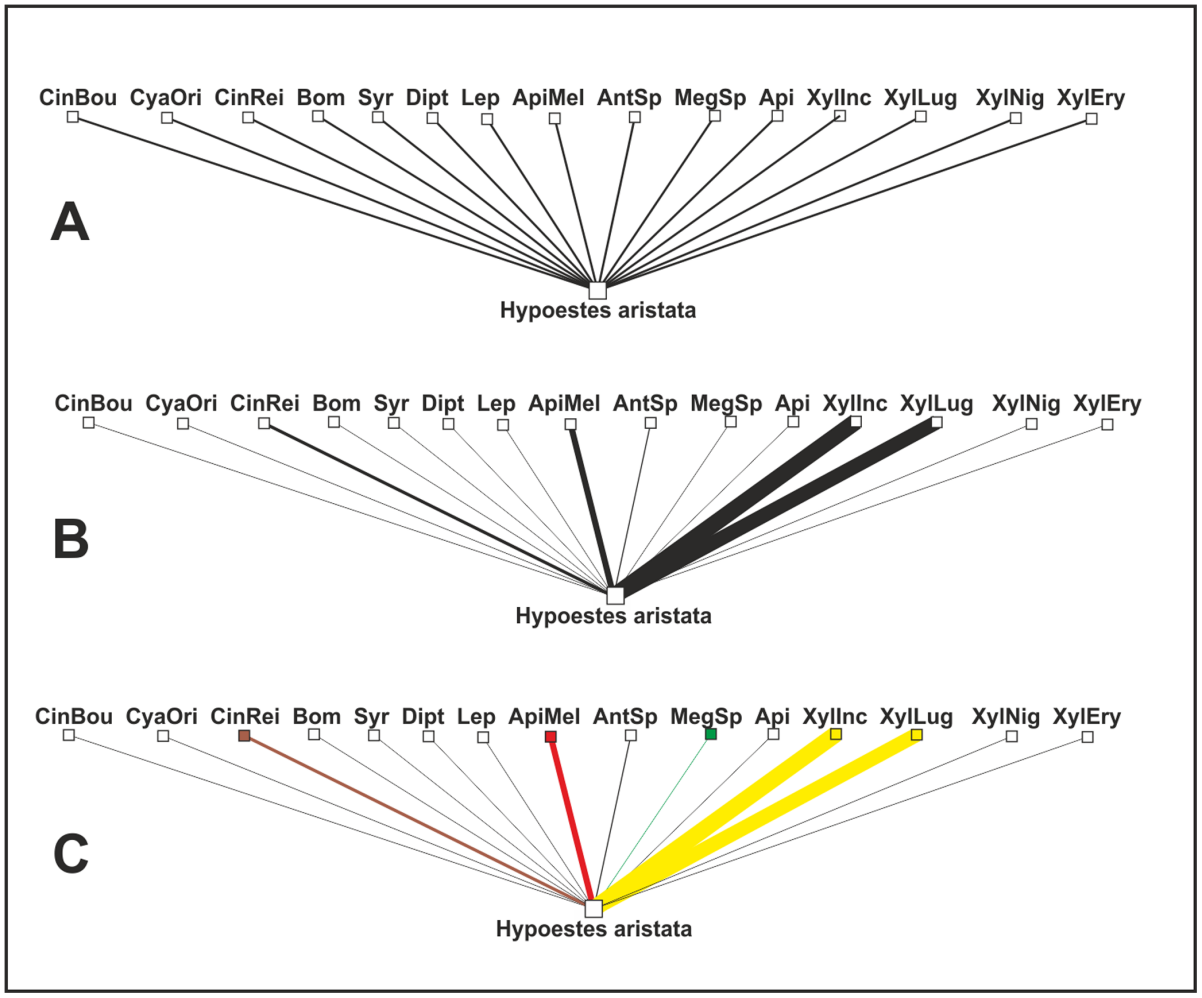
Interactions between *H. aristata* and its visitors. (A) Binary interactions showing just the visitor-plant interaction - the approach commonly used in pollination networks. (B) Quantitative interactions showing the frequencies of visits by individual visitors - the less frequently used approach in pollination networks. (C) Quantitative interactions indicating the role of individual visitors: yellow = important effective pollinators, green = pollinators with a marginal effect on *H. aristata* reproduction, red = nectar thieves with a negative impact on *H. aristata* reproduction; brown = nectar thieves with a potential negative effect on *H. aristata* reproduction; and black, visitors with no effect on *H. aristata* reproduction. Abbreviations: **CinBou** = *Cinnyris bouvieri*, **CyaOri** = *Cyanomitra oritis*, **CynRei** = *Cinnyris reichenowi*, **Bom** = Bombyliidae, **Syr** = Syrphidae, **Dipt** = other dipterans, **Lep** = Lepidoptera, **ApiMel** = *Apis mellifera*, **AntSp** = *Anthophora* sp., **MegSp** = *Megachile* sp., **Api** = other bees, **XylInc** = *Xylocopa* cf. *inconstans*, **XylLug** = *Xylocopa lugubris*, **XylNig** = *Xylocopa nigrita*, **XylEry** = *Xylocopa erythrina*.

The analyses of the pollination system of *H. aristata* show different roles for individual visitors. Our finding that the two carpenter bees were the only important pollinators among the wide spectrum of floral visitors is in accordance with the bee pollination syndrome of *H. aristata* and with the concept of pollination syndrome [4], [39]. Nevertheless, as much as successful pollination is highly dependent on ‘trait-matching’ between flowers and their visitors [24], [44], [66]–[68], we agree that the visitor’s body size plays an important role in the assessment of the pollination syndrome validity. The large bees were effective pollinators whereas the relatively smaller bee *A. mellifera* had a negative effect on *H. aristata* reproduction. This assumption supports the idea that the bee pollination syndrome should be divided further into large-bee and small-bee syndromes [73], [74]. Our results are also in accordance with the most effective pollinator principle [3], supposing that the plant traits evolved as a response to the most effective pollinators. In contrast to the expected generalization of this system, we found a high degree of specialization. This would be even more apparent if we followed the ideas of Fenster et al. [75] and considered the similarly large bees *Xylocopa* spp. and *Megachile* sp. as one functional group exerting similar selection pressures. Moreover, we also observed visitors with negative or potentially negative effects on plant reproductive success. As shown in other studies [36], these visitors can create different selection pressures on various floral traits. If they are overlooked or even considered as pollinators, then our understanding could lead to a total misinterpretation of the pollination systems. Our conclusions would be completely different if we considered all visitors as pollinators as is typical in plant-pollinator web studies (Fig. 3). It also clearly demonstrates why pollination networks frequently show flowers to be phenotypically specialized but ecologically generalized [76].

Although we assume that the progress from studies on simple pollination systems (often including just one pollinator and one plant species) to community level studies is the right direction for pollination biology, we must urge, together with other researchers [1], [77], that without any knowledge of the roles of individual visitors, we cannot confirm the validity of the pollination syndrome hypothesis, determine the degree of generalization, nor create a relevant evolutionary hypothesis.

## Supporting Information

Figure S1 **Figure of the visitation frequencies, given separately for each studied patch.**
(DOC)

File S1 **Preliminary study on the breeding system of** ***Hypoestes aristata.*** The breeding system was studied by emasculation and pollen supplementation in five experimental treatments. The results showed that the experimental treatments differed in the reproductive success of *H. aristata*; i.e. in the number and total weight of seeds per fruit. **Table A,** Results of the hand-pollination experiment done by permutation mixed models. **Fig. A,** Seed number per flower (Means and Standard Errors) of *Hypoestes aristata* in five experimental treatments.
(DOC)

Movie S1 **The video file attached shows the representative visitors of** ***Hypoestes aristata*** **while foraging for the nectar.** Shots were taken at the study site by the small hand camcorder during the field studies in 2010 and 2012. Some of the presented shots were intentionally slowed to better show the visitorś behaviour. High definition of the video file was converted to fit the size limit given by the journal.
(ZIP)
