# Supplementary material for: Generalization versus Specialization in Pollination Systems: Visitors, Thieves, and Pollinators of Hypoestes aristata (Acanthaceae)
Source: PLoS One. 2013 Apr 10;8(4):e59299. doi: 10.1371/journal.pone.0059299 (PMC3622670; doi:10.1371/journal.pone.0059299)
Supplement: Figure S1 — Figure of the visitation frequencies, given separately for each studied patch. (DOC) [file pone.0059299.s001.doc]

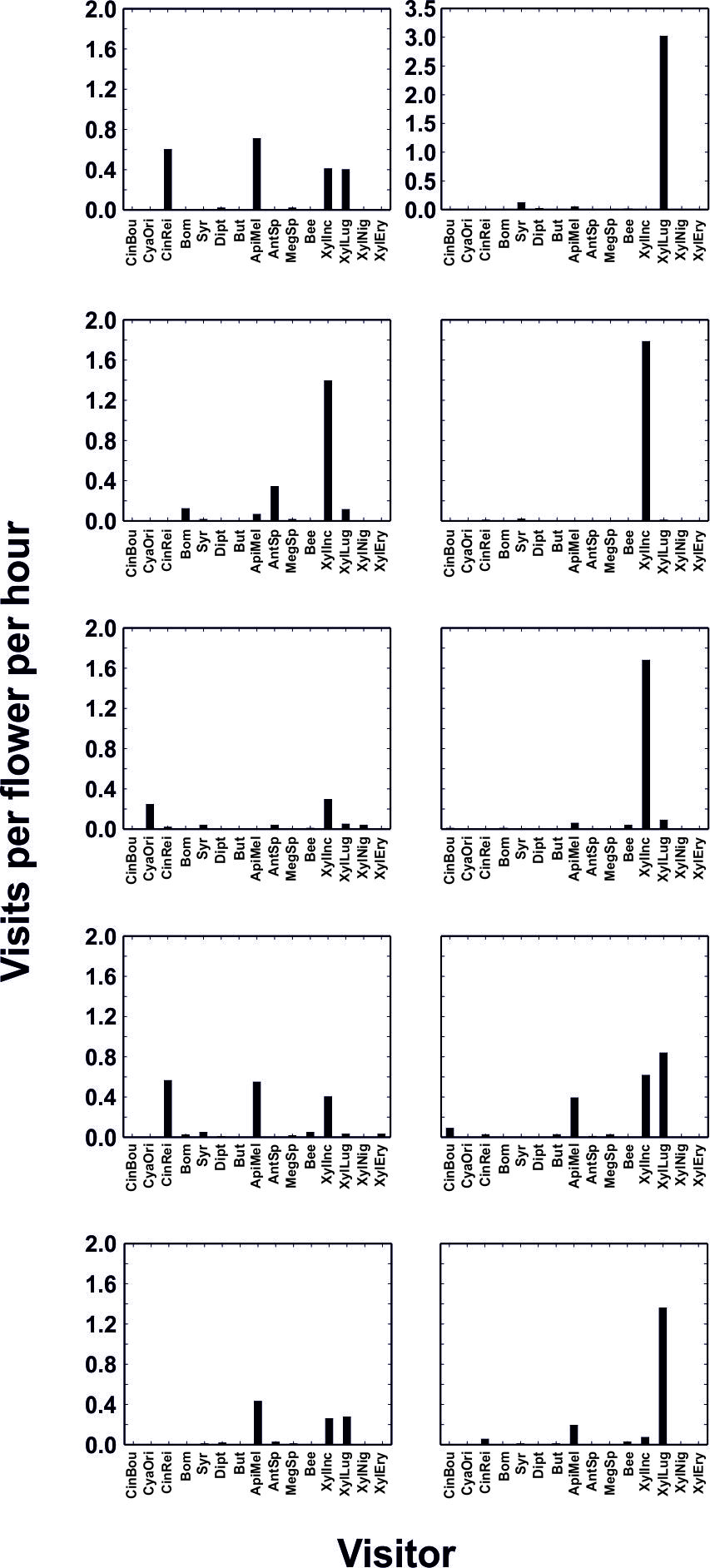


**Fig. S1**. The visitation frequencies, given separately for each studied patch. Note that the second graph has a different scale for the y-axis since *X. lugubris* was much more common than any other taxon.

Abbreviations: **CinBou** = *Cinnyris bouvieri*, **CyaOri** = *Cyanomitra oritis*, **CynRei** = *Cinnyris* *reichenowi*, **Bom** = Bombyliidae, **Syr** = Syrphidae, **Dipt** = other dipterans, **But** = Lepidoptera, **ApiMel** = *Apis mellifera*, **AntSp** = *Anthophora* sp., **MegSp** = *Megachile* sp., **Bee** = other bees, **XylInc** = *Xylocopa* cf. *inconstans*, **XylLug** = *Xylocopa lugubris*, **XylNig** = *Xylocopa* *nigrita*, and **XylEry** = *Xylocopa erythrina*.
