# Supplementary material for: Generalization versus Specialization in Pollination Systems: Visitors, Thieves, and Pollinators of Hypoestes aristata (Acanthaceae)
Source: PLoS One. 2013 Apr 10;8(4):e59299. doi: 10.1371/journal.pone.0059299 (PMC3622670; doi:10.1371/journal.pone.0059299)
Supplement: File S1 — Preliminary study on the breeding system of Hypoestes aristata. The breeding system was studied by emasculation and pollen supplementation in five experimental treatments. The results showed that the experimental treatments differed in the reproductive success of H. aristata; i.e. in the number and total weight of seeds per fruit. Table A, Results of the hand-pollination experiment done by permutation mixed models. Fig. A, Seed number per flower (Means and Standard Errors) of Hypoestes aristata in five experimental treatments. (DOC) [file pone.0059299.s002.doc]

**File S1**

**Preliminary study on the breeding system of *Hypoestes aristata***

**Methods:** The breeding system of *H. aristata* was studied by emasculation and pollen supplementation in 10 patches with more than 15 flowering shoots. In each patch, 5 shoots, each of which had 5 flowers, were randomly selected and assigned to different experimental treatments: (1) *autonomous selfing*, flowers bagged; (2) *parthenogenesis*, flowers bagged and emasculated; (3) *geitonogamy*, flowers bagged, emasculated, and hand-pollinated from flowers of the same plant; (4) *outcrossing*, flowers bagged, emasculated, and hand-pollinated by pollen from a distant population; and (5) *controls*, flowers not bagged and left to natural pollination. The shoots were bagged with a fine mesh to avoid any contact with pollinators. Treated flowers were emasculated after each flower had just opened and hand-pollinated twice a day until the stigma was active. The experimental flowers were left bagged to mature. Fruits were harvested, and seeds counted and weighed.

Because the data distribution did not fulfil the assumptions of standard ANOVA, data were analyzed by analogical permutation mixed models in the program PERMANOVA+ for PRIMER [S1]. In all models, the treatment was considered to be the fixed factor and the shoot, which was nested in the treatment, as a random factor. Differences between individual treatments were compared by post-hoc pairwise comparisons.

**Results:** The experimental treatments differed in the reproductive success of *H. aristata*; i.e. in the number and total weight of seeds per fruit. The differences in the weight of one seed were not significant (Table A). Neither autonomous selfing nor parthenogenesis guaranteed reproductive success (Fig. A). In contrast, geitonogamous, outcrossing, and control treatments resulted in significantly higher seed numbers and weight per fruit, but they did not significantly differ among each other (Fig. A).

**Reference:**

S1. Anderson, M.J., Gorley, R.N. & Clarke, K.R. (2008) *PERMANOVA+ for PRIMER: Guide to software and statistical methods. PRIMER-E: Plymouth, UK*.

**Table A**. Results of the hand-pollination experiment: Permutation mixed models.

|  |  |  | **No. of seeds per fruit** | | |  | **Weight of one seed** | | | |
| --- | --- | --- | --- | --- | --- | --- | --- | --- | --- | --- |
|  | type |  | F |  | P (perm) |  | F |  | P (perm) |  |
| **Treatment** | *fixed* |  | 5.77 |  | **0.002** |  | 1.40 |  | 0.238 |  |
| **Shoot (Treatment)** | *random* |  | 1.73 |  | **0.006** |  | 1.40 |  | 0.205 |  |

Shoot nested in treatment. Significant differences (p < 0.05) are in bold.


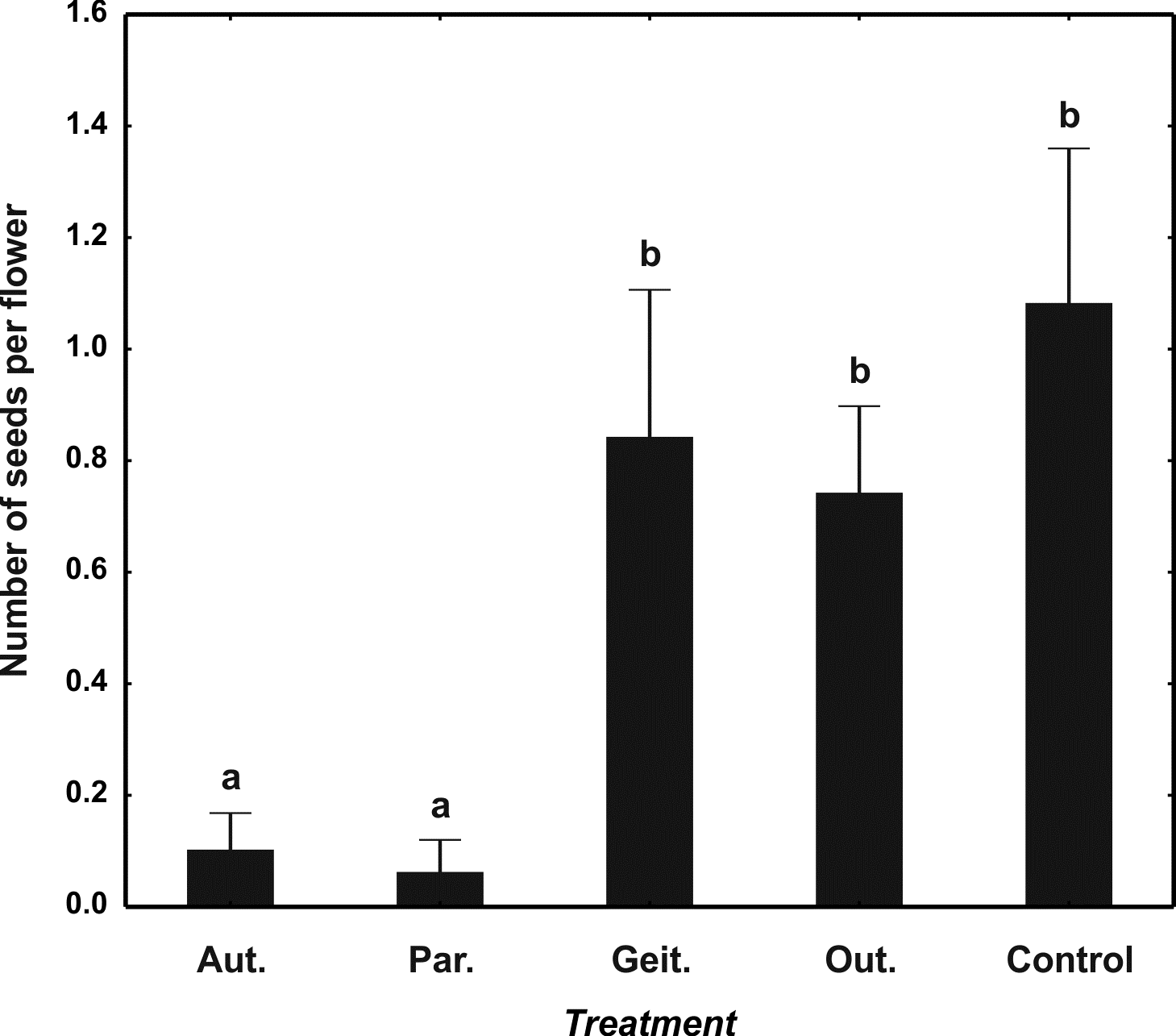


**Fig. A.** Seed number per flower (Means and Standard Errors) of *Hypoestes aristata* in five experimental treatments: ***Aut.,*** autonomous selfing; **Par.,** parthenogenesis; **Geit.,** geitonogamy; ***Out.,*** outcrossing; and ***Control***. Different letters above the boxes indicate significant differences between treatments.
